# Supplementary material for: Food Fortification of Instant Pulse Porridge Powder with Improved Iron and Zinc Bioaccessibility Using Roselle Calyx
Source: Nutrients. 2022 Sep 30;14(19):4070. doi: 10.3390/nu14194070 (PMC9573692; doi:10.3390/nu14194070)
Supplement: Supplementary file 1 [file nutrients-14-04070-s001.zip › nutrients-1930929-supplementary.pdf]

nutrients

MDPI

Supplementary materials

Food Fortification of Instant Pulse Porridge Powder with Improved Iron and Zinc Bioaccessibility Using Roselle Calyx

Sandhya Subedi, Uthaiwan Suttisansanee, Aikkarach Kettawan, Chaowanee Chupeerach, Chanakan Khemthong, Sirinapa Thangsiri and Nattira On–nom *

Food and Nutrition Academic and Research Cluster, Institute of Nutrition, Mahidol University, Salaya, Phuttamonthon, Nakhon Pathom 73170, Thailand

* Correspondence: nattira.onn@mahidol.ac.th (N.O.); Tel.: +66–(0)–2800–2380 (ext.426)

Supplementary Table S1:

Proximate, iron and zinc contents of instant porridge powders (per 100 g fresh weight).

Nutritive values (per 100 g fresh weight)	Formulations		
	**ICP**	**ICF**	**IPP**
Moisture (%)	3.96 ± 0.12 [a]	3.97 ± 0.05 [a]	6.03 ± 0.01 [b]
Energy (kcal)	440.62 ± 0.90 [c]	429.33 ± 0.62 [b]	423.50 ± 0.71 [a]
Protein (g)	19.35 ± 0.08 [c]	15.94 ± 0.11 [b]	15.50 ± 0.00 [a]
Fat (g)	14.44 ± 0.14 [c]	12.17 ± 0.22 [a]	12.90 ± 0.00 [b]
Carbohydrate (g)	58.32 ± 0.18 [a]	64.02 ± 0.23 [c]	61.45 ± 0.07 [b]
Total dietary fiber (g)	16.48 ± 0.53 [c]	11.08 ± 0.24 [a]	12.97 ± 0.00 [b]
Ash (g)	3.94 ± 0.57 [a]	3.91 ± 0.07 [a]	4.18 ± 0.01 [b]
Iron (mg) [ns]	4.08 ± 0.13	4.07 ± 0.16	4.59 ± 0.23
Zinc (mg)	2.82 ± 0.18 [a]	2.61 ± 0.13 [a]	3.71 ± 0.20 [b]

All data are shown as the mean ± standard deviation (SD) of triplicate determination (n=3). Different lowercase letters denote significantly different contents of the same proximate composition or zinc content at $p < 0.05$, while [ns] denotes no significant differences of iron content at $p \geq 0.05$ in different instant porridge powder formulations using one-way ANOVA, followed by Duncan's multiple comparison test. ICP: instant chickpea powder using pre-gelatinized chickpea flour (PCPF); ICF: instant composite flour using PCPF and pregelatinized foxtail millet flour (PFMF); IPP: instant pulse porridge powder using PCPF, PFMF and roselle calyx powder (RCP).

Supplementary Table S2:

Sensory evaluation of instant porridge powders with different amount of roselle calyx powder.

Instant Porridge Powders	Sensory attributes					
	Appearance ^{ns}	Color ^{ns}	Odor	Taste ^{ns}	Texture ^{ns}	Overall liking
IPP-4	6.73 ± 1.38	6.70 ± 1.36	7.10 ± 1.21 ^{a}	6.60 ± 1.35	6.73 ± 0.93	6.85 ± 1.10 ^{a}
IPP-6	6.75 ± 1.48	6.78 ± 1.46	6.48 ± 2.00 ^{b}	6.25 ± 1.76	6.33 ± 1.33	6.33 ± 1.64 ^{ab}
IPP-8	6.65± 1.53	6.70 ± 1.51	6.50 ± 1.36 ^{b}	5.95 ± 1.91	6.50 ± 1.36	6.00 ± 1.64 ^{b}

All data are shown as the mean ± standard deviation (SD) of 40 untrained panelists (n = 40). Different lowercase letters denote significantly different of the sensory attributes on odor and overall liking at $p < 0.05$, while ^{ns} denotes no significantly different values at $p \geq 0.05$ of the sensory attributes on appearance, color, taste and texture in different instant porridge powder formulations using one–way ANOVA, followed by Duncan's multiple comparison test. IPP-4: instant pulse porridge powder using pre-gelatinized chickpea flour (PCPF), pregelatinized foxtail millet flour (PFMF) and 4.99% (*w/w*) roselle calyx powder (RCP); IPP-6: instant pulse porridge powder using PCPF, PFMF and 6.65% (*w/w*) RCP; IPP-8: instant pulse porridge powder using PCPF, PFMF and 8.31% (*w/w*) RCP. Based on information in this table, the sensory evaluation showed better odor in IPP-4 than IPP-6 and IPP-8. Besides, no significant differnce on overall liking was observed between IPP-4 and IPP-6, while IIP-8 exhibited the significant lowest overall liking score.

Supplementary Table S3:

In vitro iron and zinc bioaccessibility of instant porridge powders with different amount of roselle calyx powder.

Formulations	% Bioaccessibility	
	Iron ^{ns}	Zinc ^{ns}
IPP-4	11.08 ± 0.86	45.83 ± 2.90
IPP-6	11.98 ± 0.20	49.70 ± 1.41

All data are shown as the mean ± standard deviation (SD) of 40 untrained panelists (n = 40). The ^{ns} denotes no significantly different values at $p \geq 0.05$ of the in vitro iron and zinc bioaccessibility in different instant porridge powder formulations using using Student's unpaired *t*-test. IPP-4: instant pulse porridge powder using pre-gelatinized chickpea flour (PCPF), pregelatinized foxtail millet flour (PFMF) and 4.99% (*w/w*) roselle calyx powder (RCP); IPP-6: instant pulse porridge powder using PCPF, PFMF and 6.65% (*w/w*) RCP. Based on information in Supplementary Table S2 and S3 with no significant differences in in vitro iron and zinc bioaccessibility between IIP-4 and IPP-6, IPP-4 was selected for this study due to lower cost with more desirable odor and taste comparing to IPP-6.
